# Supplementary material for: Cortical astrocyte N-methyl-D-aspartate receptors influence whisker barrel activity and sensory discrimination in mice
Source: Nat Commun. 2024 Feb 21;15:1571. doi: 10.1038/s41467-024-45989-3 (PMC10882001; doi:10.1038/s41467-024-45989-3)
Supplement: Supplementary file 1 — Supplementary Information [file 41467_2024_45989_MOESM1_ESM.pdf]

SUPPLEMENTARY INFORMATION

Cortical astrocyte N-Methyl-D-Aspartate receptors influence whisker barrel activity and sensory discrimination

Noushin Ahmadpour*[1], Meher Kantroo*[1], Michael J. Stobart[1], Jessica Meza-Resillas[1], Shahin Shabanipour[1], Jesus Parra Nunez[1], Tania Salamovska[1], Anna Muzaleva[1], Finnegan O'Hara[1], Dustin Erickson[1], Bruno Di Gaetano[1], Sofia Carrion-Falgarona[1], Bruno Weber[2], Alana Lamont[3,4], Natalie E. Lavine[3,4], Tiina M. Kauppinen[3,4], Michael F. Jackson[3,4], Jillian L. Stobart[1,5]

(1) College of Pharmacy, University of Manitoba, Winnipeg, MB, Canada
(2) Institute of Pharmacology and Toxicology, University of Zurich, Zurich, Switzerland
(3) Department of Pharmacology and Therapeutics, University of Manitoba, Winnipeg, MB, Canada
(4) PrairieNeuro Research Center, Health Sciences Center, Winnipeg, MB, Canada
(5) Centre on Aging, University of Manitoba, Winnipeg, MB, Canada

*** Co-first author**

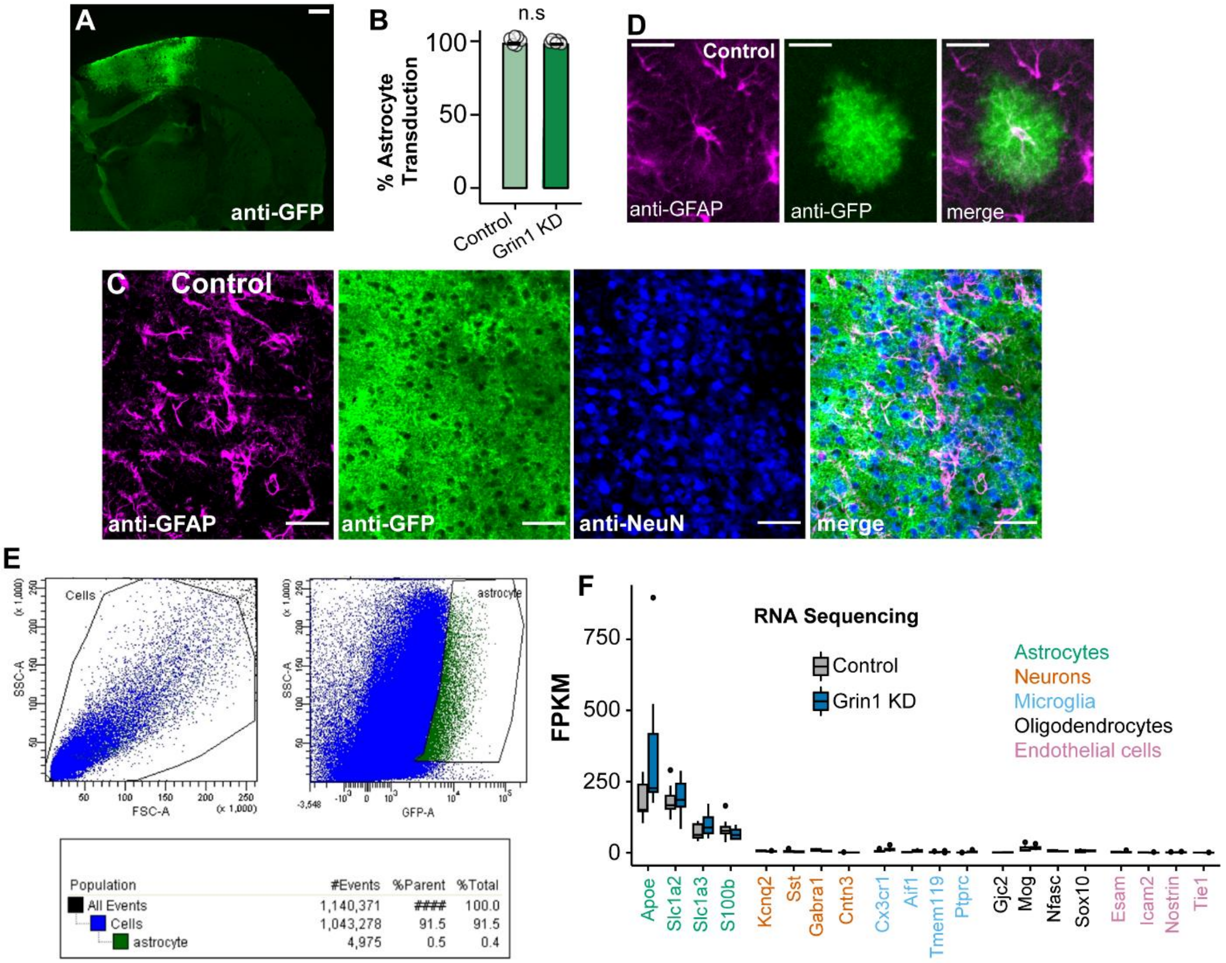

Supplementary Figure 1. Astrocyte viral transduction and FACS. A) The astrocyte viral constructs (green, stained with anti-GFP) labelled a good section of cortex. Scale bar= 500 µm. B) The viruses transduced more than 95% of GFAP positive astrocytes in the injection area. n= 6 fields of view in each group from 3 mice. Statistics were calculated by Mann-Whitney-Wilcoxon test. C,D) Lck-GCaMP6f (anti-GFP) from control AAV localized to astrocytes (anti-GFAP), but not neurons (anti-NeuN) by immunohistochemistry. Scale bar= 25 µm. E) Example dot plots from the flow cytometer with non-fluorescent cells (blue) and the collected GFP population (green). Astrocytes were selected based on GCaMP6f (GFP) fluorescence and large granularity (SSC-A). First, FSC-A/SSC-A was used to gate cells and exclude the debris. Next, the GFP-A/SSC-A gate was used to isolate the GCaMP positive cell population. F) RNA from cells sorted by FACs showed an enrichment for astrocyte markers by RNA sequencing over markers for neurons, microglia, oligodendrocytes, and endothelial cells. Data represented as fragments per kilobase of transcript per

million mapped reads (FPKM). Boxplot data includes median and quartile values. Source data are provided as a Source Data file.

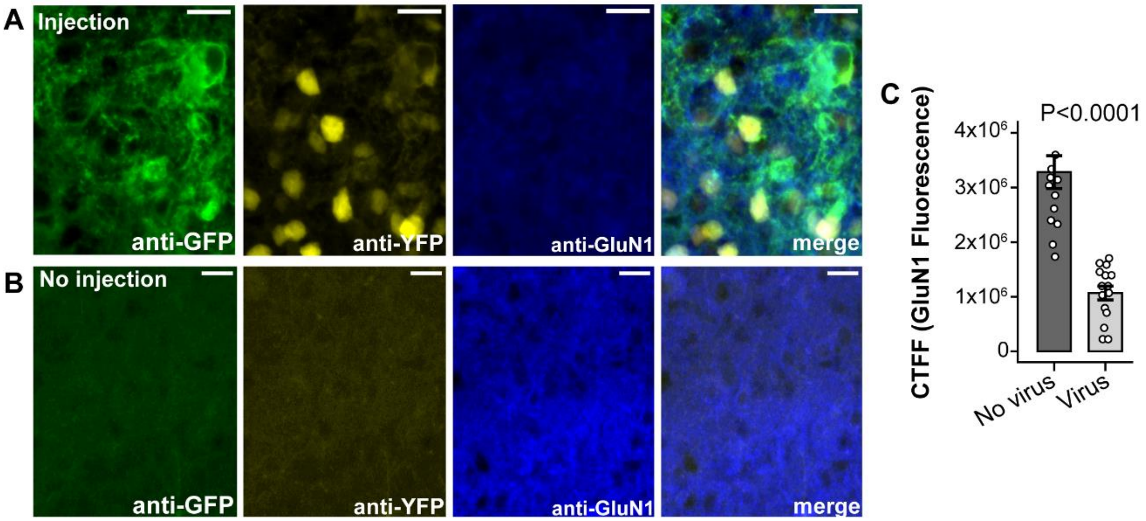

Supplementary Figure 2. Neuronal depletion of GluN1. To detect changes in astrocyte GluN1 expression by immunohistochemistry, we decreased neuronal GluN1 expression in the virus injection area by injecting a hSYN-Cre virus into floxed Grin1 mice. A) Example immunohistochemistry for GluN1 (blue) in the virus injection area. Mice also had a Cre-dependent fluorescent reporter (eYFP), so yellow neurons were identified as the depleted cells. GFP expression was localized to astrocytes expressing our control or Grin1 KD astrocytes. B) Immunohistochemistry for GluN1 (blue) outside of the virus injection area. No GFP or YFP signal was detected, particularly in neuronal somata. Scale bar= 15 µm. C) The corrected total field fluorescence (CTFF) for fields of view outside (no virus) or inside (virus) the hSYN-Cre + GFAP-control virus injections show a clear reduction in widespread GluN1 fluorescence in the virus area. n= 11 fields with no virus; n= 15 fields with virus; 3 mice. Data is mean ± SEM and dots are individual fields. Stats were performed with linear mixed models and Tukey post-hoc tests. Source data are provided as a Source Data file.

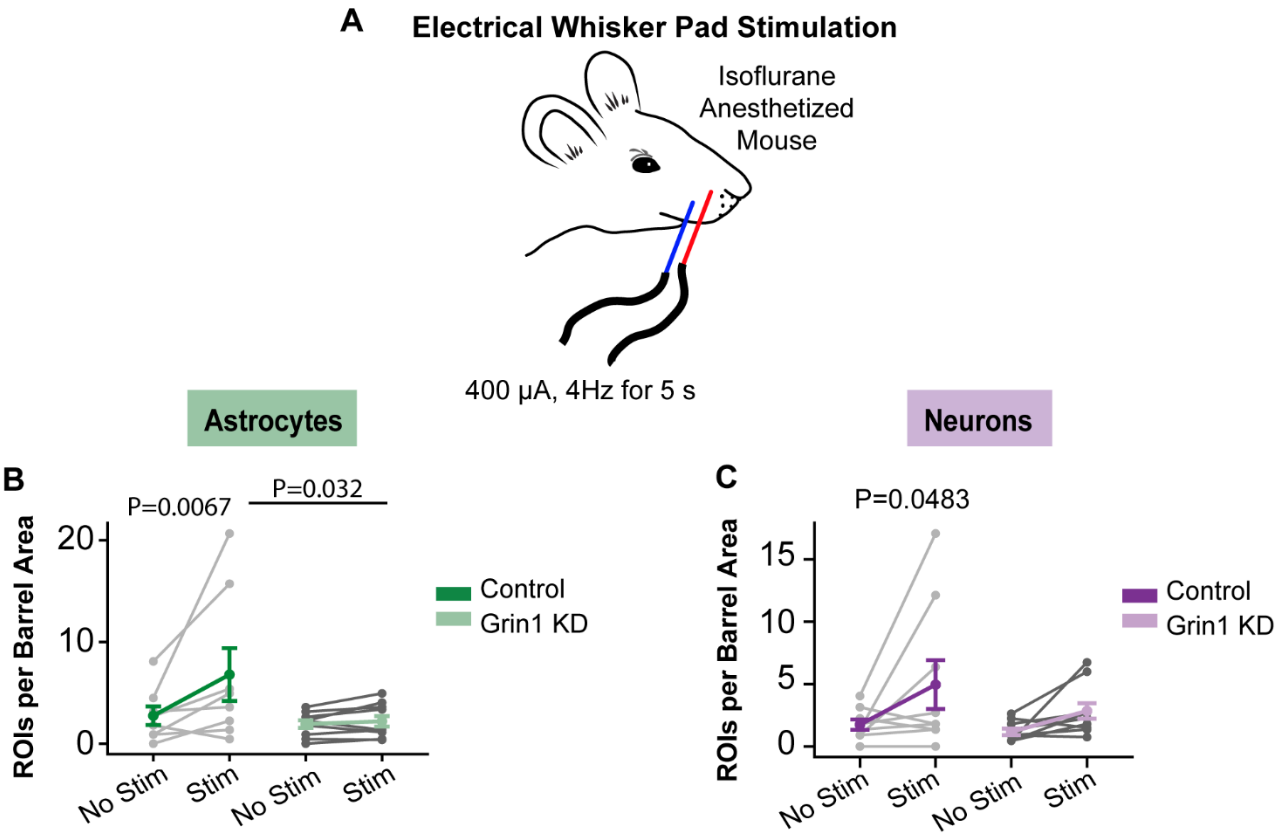

Supplementary Figure 3. Astrocyte and neuron responses to brief electrical whisker pad stimulation in anesthetized mice. A) Experimental schematic. B) Number of astrocyte microdomain ROIs per barrel area evoked by electrical stimulation. C) Number of neuron ROIs per barrel area evoked by electrical stimulation. n= 8 control and 10 Grin1 KD mice. Grey paired lines are individual fields of view. Coloured lines (green or purple) are mean ± SEM. Statistics were calculated using linear mixed model and Tukey post hoc tests. Source data are provided as a Source Data file.

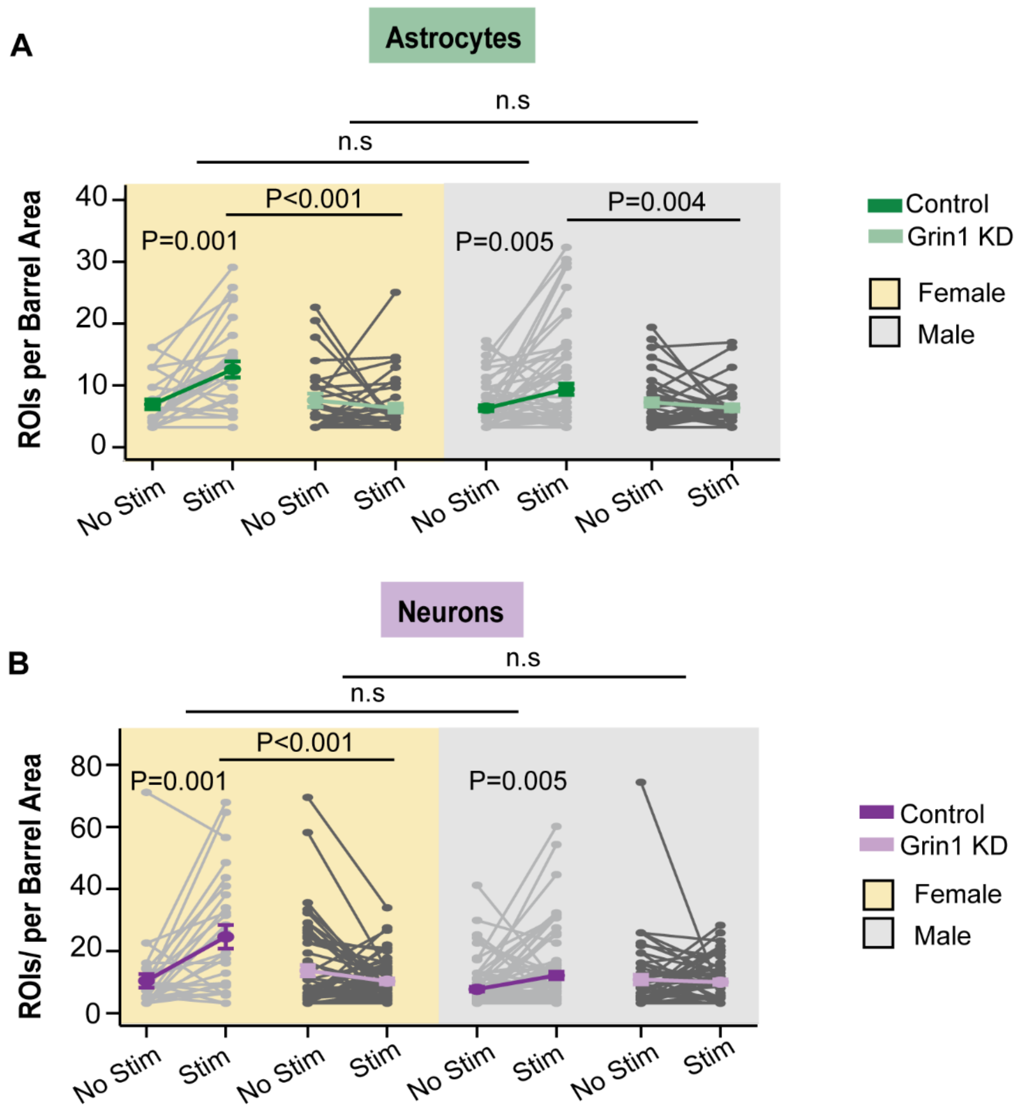

Supplementary Figure 4. Sex comparisons for astrocyte and neuron Ca^{2+} responses. A) Mean number of ROIs/barrel area in Lck-GCaMP6f astrocytes from male and female mice. B) Mean number of ROIs/barrel area in RCaMP1.07 neurons from male and female mice. No sex differences were detected in any case. Control: n=103 FOV, 4 female and 4 male, Grin1 KD: n=97 FOV, 6 female mice, 5 male mice. Grey paired lines are individual fields of view. Coloured lines (green or purple) are mean ± SEM. Statistics were calculated using linear mixed model and Tukey post hoc tests. Source data are provided as a Source Data file.

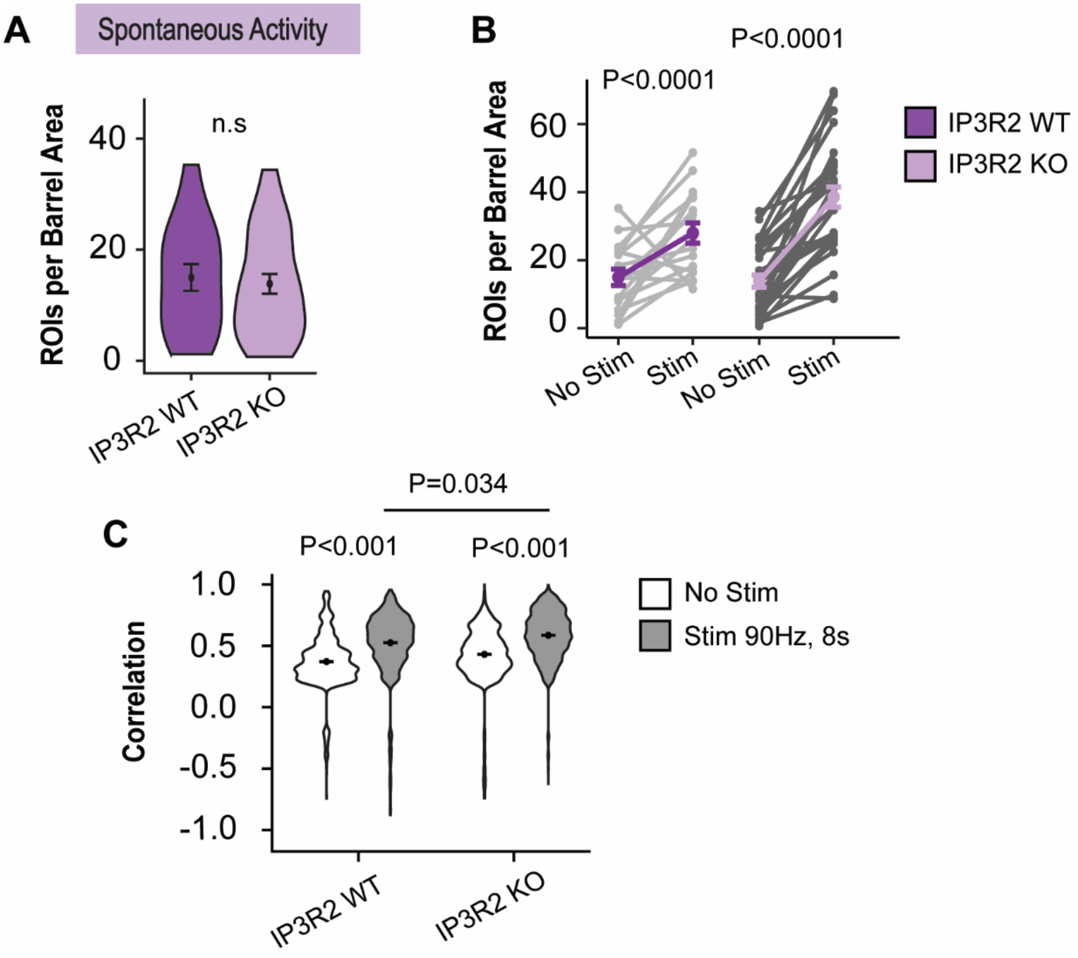

Supplementary Figure 5. Mice lacking IP3R2 (IP3R2 KO) do not show altered neuronal responses to whisker stimulation like Grin1 KD mice. Using the same awake two-photon paradigm as Grin1 KD animals, we followed neurons expressing hSYN-RCaMP1.07 in IP3R2 KO and litter mate IP3R2 wildtype (WT) control mice. Violin plots show the distribution of all ROIs or all FOVs with the mean and SEM error bars plotted on top. A) The number of spontaneous neurons (ROIs per square area) during quiet wakefulness of the animal was not different between IP3R2 WT and KOs. B) The mean number of responding neurons (ROIs per barrel area) increased during whisker stimulation in IP3R2 WT and IP3R2 KO. Grey paired lines are individual fields of view. Coloured lines (purple) are mean ± SEM. C) The Pearson's correlation between pairs of active neurons in the same FOV increased with whisker stimulation in both IP3R2 WT and IP3R2 KO. The correlation was slightly higher in IP3R2 KO than WT. Violin plots show the distribution of all neuronal correlations with the mean and SEM error bars plotted on top. n=17 FOV from 3 control mice; n=29 FOV from 4 KO mice. Statistics were calculated using linear mixed model and Tukey post hoc tests. Source data are provided as a Source Data file.

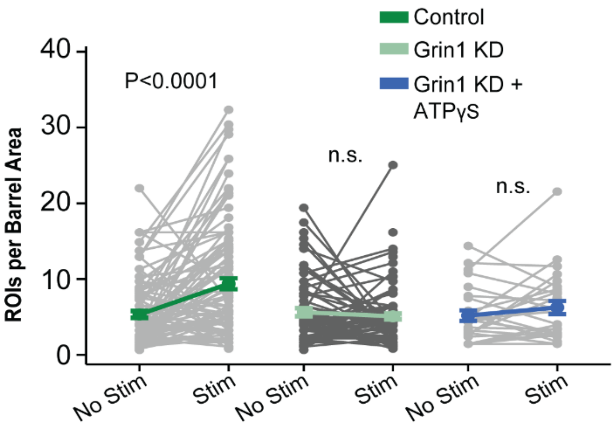

Supplementary Figure 6. ATPγS does not improve stimulus-evoked calcium signaling in Grin1 KD astrocytes. The number of astrocyte calcium microdomain ROIs responding with Ca^{2+} events during stimulation did not increase in Grin1 KD astrocytes during treatment with ATPγS. Control: N= 103 FOV in 8 mice, Grin1 KD: N= 97 FOV from 11 mice. ATPγS group: N= 54 FOVs from 10 mice. Grey paired lines are individual fields of view. Coloured lines (green or blue) are mean ± SEM. Source data are provided as a Source Data file.
