# Supplementary material for: Cortical astrocyte N-methyl-D-aspartate receptors influence whisker barrel activity and sensory discrimination in mice
Source: Nat Commun. 2024 Feb 21;15:1571. doi: 10.1038/s41467-024-45989-3 (PMC10882001; doi:10.1038/s41467-024-45989-3)
Supplement: Supplementary file 3 — Description of Additional Supplementary Files [file 41467_2024_45989_MOESM3_ESM.docx]

**Description of Additional Supplementary Files**

**Supplementary Movie 1:**  Example field of view captured by in vivo two-photon microscopy of astrocyte and neuron calcium transients from a control mouse during whisker stimulation. The timing of the whisker stimulus is indicated by the white *. Lck-GCaMP fluorescence in astrocytes (green and upper heat map) shows increased calcium events throughout the astropil during stimulation. RCaMP fluorescence in neurons (magenta and lower heat map) also increases during stimulation. The two neurons indicated with white arrows are particularly responsive to stimulation. Time stamp indicates seconds.

**Supplementary Movie 2:** Example field of view captured by in vivo two-photon microscopy of astrocyte and neuron calcium transients from a Grin1 KD mouse during whisker stimulation. The timing of the whisker stimulus is indicated by the white *. Lck-GCaMP fluorescence in astrocytes (green and upper heat map) shows fewer localized Ca2+ events in astrocytes compared to Movie 1. RCaMP fluorescence in neurons (magenta and lower heat map) shows spontaneous activity (yellow arrows) and responses to whisker stimulation (white arrow). Time stamp indicates seconds.
