# Supplementary material for: Cortical astrocyte N-methyl-D-aspartate receptors influence whisker barrel activity and sensory discrimination in mice
Source: Nat Commun. 2024 Feb 21;15:1571. doi: 10.1038/s41467-024-45989-3 (PMC10882001; doi:10.1038/s41467-024-45989-3)
Supplement: Supplementary file 6 — Reporting Summary [file 41467_2024_45989_MOESM6_ESM.pdf]

nature portfolio

Corresponding author(s): Jillian Stobart

Last updated by author(s): Feb 1, 2024

Reporting Summary

Nature Portfolio wishes to improve the reproducibility of the work that we publish. This form provides structure for consistency and transparency in reporting. For further information on Nature Portfolio policies, see our Editorial Policies and the Editorial Policy Checklist.

Statistics

For all statistical analyses, confirm that the following items are present in the figure legend, table legend, main text, or Methods section.

n/a	Confirmed	
☐	☒	The exact sample size (*n*) for each experimental group/condition, given as a discrete number and unit of measurement
☐	☒	A statement on whether measurements were taken from distinct samples or whether the same sample was measured repeatedly
☐	☒	The statistical test(s) used AND whether they are one- or two-sided<br>*Only common tests should be described solely by name; describe more complex techniques in the Methods section.*
☐	☒	A description of all covariates tested
☐	☒	A description of any assumptions or corrections, such as tests of normality and adjustment for multiple comparisons
☐	☒	A full description of the statistical parameters including central tendency (e.g. means) or other basic estimates (e.g. regression coefficient) AND variation (e.g. standard deviation) or associated estimates of uncertainty (e.g. confidence intervals)
☐	☒	For null hypothesis testing, the test statistic (e.g. *F*, *t*, *r*) with confidence intervals, effect sizes, degrees of freedom and *P* value noted<br>*Give P values as exact values whenever suitable.*
☒	☐	For Bayesian analysis, information on the choice of priors and Markov chain Monte Carlo settings
☐	☒	For hierarchical and complex designs, identification of the appropriate level for tests and full reporting of outcomes
☐	☒	Estimates of effect sizes (e.g. Cohen's *d*, Pearson's *r*), indicating how they were calculated

Our web collection on statistics for biologists contains articles on many of the points above.

Software and code

Policy information about availability of computer code

Data collection	Two-photon images were collected using PrairieView (v5.4); a software that controls all of the scanning and image collection functions of Ultima In Vivo/In Vitro two-photon laser-scanning microscope.<br>Animal behavior setup was controlled by a custom-designed MATLAB (R2020b) algorithm.
Data analysis	Calcium analysis was done using CHIPS (v1.1.0); an open-source MATLAB toolbox n open-source MATLAB toolbox designed to analyse functional images of cells and blood vessels, primarily from two-photon microscopy. CHIPS is available at //github.com/EIN-lab/CHIPS/releases.<br>Data analysis was done using R Studio (v1.2.1335); an integrated development environment for R, a programming language for statistical computing and graphics.<br>Behavior data analysis was partly done using DeepLabCut (v2.0); Markerless pose estimation of user-defined features with deep learning. DeepLabCut is available at //github.com/AlexEMG/DeepLabCut<br>Immunocytochemistry and immunohistochemistry analysis was done in Fiji (ImageJ).

For manuscripts utilizing custom algorithms or software that are central to the research but not yet described in published literature, software must be made available to editors and reviewers. We strongly encourage code deposition in a community repository (e.g. GitHub). See the Nature Portfolio guidelines for submitting code & software for further information.

Data

Policy information about availability of data

All manuscripts must include a data availability statement. This statement should provide the following information, where applicable:

- Accession codes, unique identifiers, or web links for publicly available datasets
- A description of any restrictions on data availability
- For clinical datasets or third party data, please ensure that the statement adheres to our policy

Requests for data should be directed to and will be fulfilled by Dr. Jill Stobart (jillian.stobart@umanitoba.ca) upon publication of this manuscript.

Human research participants

Policy information about studies involving human research participants and Sex and Gender in Research.

Reporting on sex and gender	N/A
Population characteristics	N/A
Recruitment	N/A
Ethics oversight	N/A

Note that full information on the approval of the study protocol must also be provided in the manuscript.

Field-specific reporting

Please select the one below that is the best fit for your research. If you are not sure, read the appropriate sections before making your selection.

[x] Life sciences [ ] Behavioural & social sciences [ ] Ecological, evolutionary & environmental sciences

For a reference copy of the document with all sections, see nature.com/documents/nr-reporting-summary-flat.pdf

Life sciences study design

All studies must disclose on these points even when the disclosure is negative.

Sample size	Before starting this study, we conducted a sample size calculation for linear mixed models (smpsize_lmm) with the "sjstats" package in R. We input a moderate effect size of 0.2, a power of 0.8, and a significance level of 0.05. Assuming 3 cluster groups (animals) and at least 3 imaging sessions per animal, a total sample size of 127 regions of interest were calculated to be required. All data in our study included larger sample sizes (more animals and regions of interest).
Data exclusions	For Calcium imaging in awake mice, images with extreme animal movement (that could not be corrected by CHIPS) were excluded as locomotion induces confounding large global calcium waves. It also changes the focal plane and/or field of view.<br>In two-photon calcium imaging of brain slices, data with severe drift were excluded as the movement changes the focal plane/field of view, creating noise.<br>In behavior tests, one animal showing signs of stress, i.e. not investigating the arena or the objects, was excluded.<br>In RNAseq, Two samples from the Grin1 KD group and 1 sample from the control group were excluded from the differential gene analysis because a) these samples came out differently than other samples in their group during a PCA analysis of the RNA sequencing results, b) they did not perform as expected during qPCR (higher or lower Grin1 expression than other samples in the same group) which could suggest variability from previous preparation steps (FACS, etc.), and c) there was very little RNA remaining after qPCR to be sent for sequencing, resulting in fewer sequencing reads for these samples.
Replication	All data was collected from multiple small cohorts of animals that were subjected to imaging and behaviour at the same time. In case of immunocytochemistry, three replicates of glial cultures were used. Imaging was performed in control and experimental groups of all three replicates. We found the data was reproducible across individual animals and cohorts.
Randomization	Wildtype mice (C57BL6/N) of each sex were ordered in equal numbers with 5 mice per cage. Animals from each cage were selected at random by the experimenter at the time of virus injection surgery and placed in a new cage based on their virus injection group (control or Grin1 knockdown; 2-3 animals per group from each cage).
Blinding	Behavior data was acquired by the same experimenter who had done the surgical procedures and was aware of the experimental groups. However, for behavior data analysis, researchers were blind to the animal group they were analyzing.<br>Blinding was not possible during imaging experiments because there were clear differences within the recorded images that disclosed the groups to the experimenter. Analysis of imaging data was done with automated software and standardized parameters without experimenter

blinding.

Reporting for specific materials, systems and methods

We require information from authors about some types of materials, experimental systems and methods used in many studies. Here, indicate whether each material, system or method listed is relevant to your study. If you are not sure if a list item applies to your research, read the appropriate section before selecting a response.

Materials & experimental systems

n/a	Involved in the study
☐	☒ Antibodies
☒	☐ Eukaryotic cell lines
☒	☐ Palaeontology and archaeology
☐	☒ Animals and other organisms
☒	☐ Clinical data
☒	☐ Dual use research of concern

Methods

n/a	Involved in the study
☒	☐ ChIP-seq
☐	☒ Flow cytometry
☒	☐ MRI-based neuroimaging

Antibodies

Antibodies used	Chicken-anti-GFP (1:1000); Aves, product# GFP-1020, Lot# GFP879484<br>Rabbit-anti-GFAP (1:3000); Dako, product# Z0334, Lot# 20059062<br>Mouse-anti-NeuN (1:200); Millipore, product# MAB377, Lot#3612227<br>Mouse-anti-GluN1 (1:200); Millipore, product# MAB363, Lot#3985576<br><br>Donkey-anti-Chicken-IgG-Alexa488 (1:1000); Invitrogen, product# A-78948<br>Donkey-anti-Rabbit-IgG-Alexa568 (1:1000); Invitrogen, product# A-10042<br>Donkey-anti-Mouse-IgG-Alexa647 (1:1000); Invitrogen, product# A-31571
Validation	These primary antibodies have been published repeatedly. For example, see Stobart et al. 2018 Neuron. and Macheler et al. 2016 Cell Metabolism.

Animals and other research organisms

Policy information about studies involving animals; ARRIVE guidelines recommended for reporting animal research, and Sex and Gender in Research

Laboratory animals	C57BL/6NCrl mice injected with AAV at 3-5 months, and used for data collection at 5 to 9 months of age.<br>Grin1fl/fl (B6.129 Grin1tm2Stl/tm2Stl; Jax #00524634) crossed with EYFPfl/fl (Gt(ROSA)26Sortm1(EYFP); Jax #00614835) mice<br>IP3R2 WT and IP3R2 KO mice (Itpr2tm1Chen)
Wild animals	The study did not involve wild animals.
Reporting on sex	Male and female mice were included in the study. For in vivo calcium analysis, we considered sex as a fixed effect however, we did not observe any sex differences.
Field-collected samples	The study did not involve samples collected from the field.
Ethics oversight	All experimental procedures were approved by the Animal Care Committee of the University of Manitoba in accordance with the Canadian Council on Animal Care.

Note that full information on the approval of the study protocol must also be provided in the manuscript.

Flow Cytometry

Plots

Confirm that:

☒ The axis labels state the marker and fluorochrome used (e.g. CD4-FITC).

☒ The axis scales are clearly visible. Include numbers along axes only for bottom left plot of group (a 'group' is an analysis of identical markers).

☒ All plots are contour plots with outliers or pseudocolor plots.

☒ A numerical value for number of cells or percentage (with statistics) is provided.

Methodology

Sample preparation	Samples were collected from the injected areas of cortices. Samples were then incubated with Dispase II, gently dissociated and strained using a 40µm pipette tip cell strainer. Cells were then washed with ice-cold HBSS+Ca+Mg and finally, the pellet was gently resuspended in ice-cold HBSS+Ca+Mg containing DNaseI (5U; Fisher Scientific; RQ1) and placed on ice until FACS.
Instrument	BD FACSAriaIII
Software	FACSDiva (Version 6.1.3) was used for data collection.
Cell population abundance	5,000-15,000 GCaMP positive cells were collected in each run.
Gating strategy	*Describe the gating strategy used for all relevant experiments, specifying the preliminary FSC/SSC gates of the starting cell population, indicating where boundaries between "positive" and "negative" staining cell populations are defined.*

☒ Tick this box to confirm that a figure exemplifying the gating strategy is provided in the Supplementary Information.
